# Context Transfer in Reinforcement Learning Using Action-Value Functions

**DOI:** 10.1155/2014/428567

**Published:** 2014-12-31

**Authors:** Amin Mousavi, Babak Nadjar Araabi, Majid Nili Ahmadabadi

**Affiliations:** ^1^Cognitive Robotics Lab, Control and Intelligent Processing Center of Excellence, School of Electrical and Computer Engineering, College of Engineering, University of Tehran, P.O. Box 14395-515, Tehran, Iran; ^2^School of Cognitive Science, Institute for Research in Fundamental Sciences (IPM), P.O. Box 19395-5746, Tehran, Iran

## Abstract

This paper discusses the notion of context transfer in reinforcement learning tasks. Context transfer, as defined in this paper, implies knowledge transfer between source and target tasks that share the same environment dynamics and reward function but have different states or action spaces. In other words, the agents learn the same task while using different sensors and actuators. This requires the existence of an underlying common Markov decision process (MDP) to which all the agents' MDPs can be mapped. This is formulated in terms of the notion of MDP homomorphism. The learning framework is *Q*-learning. To transfer the knowledge between these tasks, the feature space is used as a translator and is expressed as a partial mapping between the state-action spaces of different tasks. The *Q*-values learned during the learning process of the source tasks are mapped to the sets of *Q*-values for the target task. These transferred *Q*-values are merged together and used to initialize the learning process of the target task. An interval-based approach is used to represent and merge the knowledge of the source tasks. Empirical results show that the transferred initialization can be beneficial to the learning process of the target task.

## 1. Introduction

The notion of transfer learning is a challenging area in the field of reinforcement learning (RL) [1–3]. The goal is to accelerate the learning process of a target task by an agent by using the knowledge of a different agent that has already learned a related task. Lazaric [1] classifies the transfer problems in RL into three categories: goal, dynamics, and domain transfer problems. A goal transfer problem is a problem in which agents share the same context (i.e., state and action spaces) and the same transition model but have different reward functions. A dynamics transfer problem is a problem in which agents share the same context and the same reward function but have different transition models. In the case of domain transfer, the agents may have different dynamics, goals, and state-action spaces. This is the most general and complex problem of transfer.

Taylor and Stone [4] discuss another category of problems in which the agents have different representations. They referred to it as representation transfer. In this paper, the agents are assumed to have different contexts (state and action spaces). In other words, the agents act in the same environment with the same reward function even as their states and actions are different.

For example, consider two learning robots acting in the same grid world problem. The first robot uses the global positioning system (GPS) sensor and the second robot uses the proximity sensor to represent their locations. So, every location of the grid is represented by the robots, differently. The robots may use different actuators, as well. In this paper, the problem of knowledge transfer between such agents is called* context transfer*. This is formulated and discussed using the notion of Markov decision process (MDP) homomorphism [5, 6].

We use the feature spaces of the tasks as a translator between them. We assume that there is a partial one-to-many mapping between the features of the tasks. An interval-based approach is used to represent, transfer, and merge the knowledge of the source tasks.

In Section 2, the context transfer problem is formally formulated and discussed. The importance and applications of the problem are described in Section 3. The mappings between feature spaces are discussed in Section 4. The knowledge fusion and transfer method is explained in Section 5. Two case studies and results are discussed in Section 7.  Section 8 contains a brief conclusion.

## 2. Context Transfer Problem

Context transfer, as defined in this section, is the problem of knowledge transfer between agents that are in the same environment doing the same task even as their state-action spaces are different. This is because the agents may use different sets of sensors or actuators. There may also be some agents using the same set of sensors although their encoding and representation of sensory information are different. In practical domains, the encoding of the sensory information is usually redundant, as one does not have access to a minimal representation of the states. In this case, some agents may have different state spaces and models of the same environment. We will discuss this problem in terms of the notion of MDPs.

An MDP is a model of an agent's interaction with the environment [7]. We limit the discussion to discrete state-action RL agents and formulate the problem in terms of the notion of finite-state MDP homomorphism. In the case of continuous state-action agents, the problem is more complex and cannot be modeled in terms of MDP homomorphism. This will be a challenging problem and invokes a completely different approach, which is out of scope of this paper. To formulate the problem, firstly, the notion of MDP and its elements are reviewed and discussed.

**Definition 1 .**

An MDP is a tuple 〈*S*, *A*, *P*, *r*〉, where *S* is the set of all states, *A* is the set of all actions, *P* : *S* × *A* × *S* → [0,1] is the transition probability function, and *r* : *S* × *A* → *R* is the reward function.

At each time step, *t*, the agent senses the environment's state, *s*_*t*_ ∈ *S*, and performs an action, *a*_*t*_ ∈ *A*. As a consequence of its action, the agent receives a numerical reward, *r*_*t*+1_ ∈ *R*, and finds itself in a new state *s*_*t*+1_. The objective of the agent is to learn a policy for acting, *π* : *S*_*t*_ → *A*_*t*_, in order to maximize its cumulative reward.

To discuss different kinds of RL knowledge transfer problems, Lazaric [1] defines three elements of an MDP.

**Definition 2 .**

A task *T*_*i*_ is an MDP defined by the tuple 〈*S*_*i*_, *A*_*i*_, *P*_*i*_, *r*_*i*_〉, in which the state and action spaces define the context, the transition model *P*_*i*_ defines the dynamics, and the reward function *r*_*i*_ defines the goal.

The problem of knowledge transfer is defined as follows.

**Definition 3 .**

Let *T* = {*T*_1_, *T*_2_,…, *T*_*l*_} be a family of tasks, and some knowledge is gained in the learning of tasks *T*_1_, *T*_2_,…, *T*_*l*−1_. The problem of knowledge transfer is to use this knowledge to improve the learning of task *T*_*l*_.

These elements are used to classify the knowledge transfer problems [1].

**Definition 4 .**

Goal transfer is a problem in which all the tasks of *T* share the same context (i.e., state and action space) and the same transition model. Dynamics transfer is a problem in which tasks share the same context and the same reward function. In the case of domain transfer, the agents may have different dynamics, goals, and contexts.

In this paper, we define another category of transfer problems called* context transfer*; the tasks of *T* share the same dynamics and reward but have different contexts. In fact, this requires the existence of an underlying common MDP to which all the agents' MDPs can be mapped. This can be explained using the notion of MDP homomorphism [5, 6].

**Definition 5 .**

An MDP homomorphism *h* from an MDP *T* = 〈*S*, *A*, *P*, *r*〉 to an MDP *T*′ = 〈*S*′, *A*′, *P*′, *r*′〉 is a surjection *h* : Ψ → Ψ′, Ψ = *S* × *A*, and Ψ′ = *S*′ × *A*′, defined by the tuple of surjections 〈*f*, {*g*_*s*_∣*s* ∈ *S*}〉, with *h*(*s*, *a*) = (*f*(*s*), *g*_*s*_(*a*)), where *f* : *S* → *S*′ and *g*_*s*_ : *A* → *A*′, such that

(1) P′fs,gsa,fs′  =∑s′′∈[s′]Bh ∣ SPs,a,s′′, ∀s,s′∈S, a∈A

(2) $$\begin{array}{c} r^{\prime }\left(f\left(s\right),g_{s}\left(a\right)\right)=r\left(s,a\right). \end{array}$$

As *h* is a surjection, it induces a partition on Ψ denoted by *B*_*h*_, and [(*s*_1_, *a*_1_)]_*B*_*h*__ denotes the block of *B*_*h*_ to which (*s*_1_, *a*_1_) belongs, such that

(3) lllllllllllllllllllllllllllllllllllllll∀s1,a1,s2,a2∈S×A;s1,a1Bh=s2,a2Bh⟺hs1,a1=hs2,a2.

*B*_*h*∣*S*_ is the projection of *B*_*h*_ on *S*, which is a partition on *S*, and [*s*′]_*B*_*h*∣*S*__ is the block containing *s*′; for every *s*_1_, *s*_2_ ∈ *S*, [*s*_1_]_*B*_*h*∣*S*__ = [*s*_2_]_*B*_*h*∣*S*__ if and only if every block of *B*_*h*_ containing a pair in which *s*_1_(*s*_2_) is a component also contains a pair in which *s*_2_(*s*_1_) is a component.

We call *T*′ the* homomorphic image* of *T* under *h*. From condition (1) we can see that state-action pairs that have the same image under *h* have the same block transition behavior in *T*, that is, the same probability of transiting to any given block of states with the same image under *f*. Condition (2) says that state-action pairs that have the same image under *h* have the same expected reward. These conditions mean that *T*′ preserves the dynamics and rewards of *T* eliminating some of the details of the original task *T*.

Now, the notion of context transfer is defined in terms of MDP homomorphism.

**Definition 6 .**

The tasks of *T* are assumed to have the same environment's dynamics and reward function, if there is a task *T*′ = 〈*S*′, *A*′, *P*′, *r*′〉 and *l* homomorphisms *h*_1_, *h*_2_,…, *h*_*l*_ so that one of these conditions are met: (i) *T*′ is an homomorphic image of *T*_*k*_ under *h*_*k*_ and *k* ∈ {1,2,…, *l*} or (ii) *T*_*k*_ is an homomorphic image of *T*′ under *h*_*k*_ and *k* ∈ {1,2,…, *l*}. These tasks are called context transferable, and the problem of knowledge transfer on *T* is called context transfer.

In other words, the tasks are context transferable, if there is a task *T*′ where all the tasks are homomorphic images of task *T*′, or task *T*′ is a homomorphic image of all the tasks. To explain the relation of the tasks of *T*, consider the following Definition and Theorem [6].

**Definition 7 .**

State-action pairs (*s*_1_, *a*_1_) and (*s*_2_, *a*_2_) ∈ Ψ are equivalent if homomorphism *h* of *T* exists such that *h*(*s*_1_, *a*_1_) = *h*(*s*_2_, *a*_2_). States *s*_1_ and *s*_2_ ∈ *S* are equivalent if (i) for every action *a*_1_ ∈ *A*, there is an action *a*_2_ ∈ *A* such that (*s*_1_, *a*_1_) and (*s*_2_, *a*_2_) are equivalent, and (ii) for every action *a*_2_ ∈ *A*, there is an action *a*_1_ ∈ *A*, such that (*s*_1_, *a*_1_) and (*s*_2_, *a*_2_) are equivalent.

The notion of equivalence leads us to the following theorem on optimal value equivalence.

**Theorem 8 .**

Let *M*′ = 〈*S*′, *A*′, *P*′, *r*′〉 be the homomorphic image of the MDP *M* = 〈*S*, *A*, *P*, *r*〉 under *h*. For any (*s*, *a*) ∈ Ψ, *Q* ^⋆^(*s*, *a*) = *Q* ^⋆^(*h*(*s*, *a*)), where *Q* ^⋆^ is the optimal action value function. In fact, the homomorphism *h*_*i*_ induces the partition *B*_*h*_*i*__ on Ψ_*i*_ as explained before. This partition actually encodes the redundancy in the representations of states and actions of task *T*_*i*_.  Theorem 8 states that if (*s*_1_, *a*_1_), (*s*_2_, *a*_2_) ∈ Ψ_*i*_, and *h*_*i*_(*s*_1_, *a*_1_) = *h*_*i*_(*s*_2_, *a*_2_), then *Q* ^⋆^(*s*_1_, *a*_1_) = *Q* ^⋆^(*s*_2_, *a*_2_) = *Q* ^⋆^(*h*_*i*_(*s*_1_, *a*_1_)). It means that the elements of a block of the partition *B*_*h*_*i*__ have the same optimal *Q*-value, which is equal to an optimal *Q*-value of the task *T*′. It concludes that for every (*s*, *a*) ∈ Ψ_*l*_ there exists a (*s*_1_, *a*_1_) ∈ Ψ_*i*_ where *Q* ^⋆^(*s*, *a*) = *Q* ^⋆^(*s*_1_, *a*_1_) and vice versa. Let *T*_*i*_ be a source task. Consider the following definition.

**Definition 9 .**

A partition *K*_*i*_ on Ψ_*i*_ = *S*_*i*_ × *A*_*i*_ is said to be *Q*-value respecting if for (*s*_1_, *a*_1_), (*s*_2_, *a*_2_) ∈ Ψ_*i*_ and (*s*_1_, *a*_1_) ≡_*K*_*i*__ (*s*_2_, *a*_2_) implies *Q* ^⋆^(*s*_1_, *a*_1_) = *Q* ^⋆^(*s*_2_, *a*_2_).

In other words, the blocks of a *Q*-value respecting partition on Ψ_*i*_ = *S*_*i*_ × *A*_*i*_ have the same optimal *Q*-values. The set of all blocks of this partition is denoted by Ψ_*i*_/*K*_*i*_. Let *C* ∈ Ψ_*i*_/*K*_*i*_ be a block of partition *K*_*i*_. The corresponding *Q*-value of this block is denoted by *Q*_*C*_^⋆^, where

(4) $$\begin{array}{c} Q_{C}^{⋆}=Q^{⋆}\left(s_{1},a_{1}\right);\;\left(s_{1},a_{1}\right)\in C. \end{array}$$

The set of all optimal *Q*-values of task *T*_*i*_ is denoted by

(5) $$\begin{array}{c} Q_{i}^{⋆}=\left\{Q_{C}^{⋆}\;|\;C\in \frac{\Psi_{i}}{K_{i}}\right\}. \end{array}$$

This is an immediate corollary of Theorem 8.

**Corollary 10 .**

If the tasks of *T* are context transferable, then *forall* *i*, *j* ∈ {1,2,…, *l*}

(6) $$\begin{array}{c} Q_{i}^{⋆}=Q_{j}^{⋆}. \end{array}$$

The proof is straightforward. As all the MDPs of *T* have the same homomorphic image *T*′ or are homomorphic images of *T*′, then all of them have the same set of optimal *Q*-values as that of *T*′. Therefore, the sets of optimal *Q*-values are the same for all tasks.

This corollary states that the optimal *Q*-values of the source tasks can be used by the target task to accelerate the learning. In the context transfer problem, we assume that the homomorphisms *h*_1_, *h*_2_,…, *h*_*l*_ are not given, and we do not know the exact equivalent relation between state-action pairs of different tasks. Instead, we use a partial one-to-many mapping between the features of the target and source tasks to transfer the knowledge. This knowledge is expressed and combined using some intervals on *Q*-values. The following example clarifies the problem of context transfer.

**Example 11 .**

**Figure 1:**
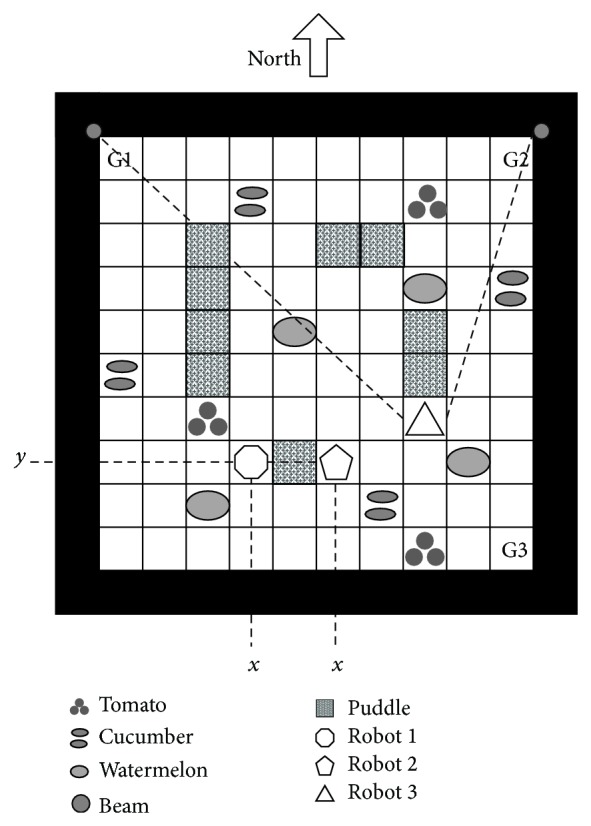
A 10 × 10 grid as a farm with three crops and three harvesting robots. Robot 1: Sensor modules: GPS, color&weight sensor, *S*_1_ = {(*x*, *y*, *k*) | 1 ≤ *x*,  *y* ≤ 10,  *k* ∈ {*RL*, *GL*, *GH*, *YH*, 0}}, *x*: column number, *y*: row number, *R*: Red, *G*: Green, *Y*: Yellow, *L*: Light, *H*: Heavy, 0: Nothing, *A*_1_ = {*N*, *S*, *E*, *W*, 0, *P*, *D*}, *N*: Move North, *S*: Move South, *E*: Move East, *W*: Move West, 0: Nothing, *P*: Pickup, *D*: Dropoff. Robot 2: Sensor modules: GPS, Compass, B&W camera, *S*_2_ = {(*x*, *y*, *d*, *c*) | 1 ≤ *x*,  *y* ≤ 10,  *d* ∈ {*N*, *S*, *E*, *W*}, *c* ∈ {SG, *T*, BG, 0}}, *x*, *y* are the same as robot 1, *d*: direction, SG: Small Globe, *T*: Rod, BG: Big Globe, 0: Nothing, *A*_2_ = {*F*, *B*, *L*, *R*, *LF*, *RF*, 0, *P*, *D*}, *F*: Move Forward, *B*: Move Backward, *L*: Turn left, *R*: Turn Right, *LF*: Turn left & *F*, *RF*: Turn right & *F*, 0: Nothing, *P*: Pickup, *D*: Dropoff. Robot 3: Sensor modules: beam's signal distance indicator, Compass, color & weight sensor, *S*_3_ = {(*b*_1_, *b*_2_, *d*, *k*) | 1 ≤ *b*_1_,  *b*_2_ ≤ 20,  *d* ∈ {*N*, *S*, *E*, *W*}, *k* ∈ {*RL*, *GL*, *GH*, *YH*, 0}}, *b*_*i*_: 1-norm distance to beam *i*, *d* is the same as robot 2 and *k* as robot 1, *A*_3_ = *A*_2_.

Consider a 10 × 10 grid as a farm with three different crops; tomato, cucumber, and watermelon (Figure 1). There are three harvesting robots that are collecting crops and gathering them into three different goal locations; tomatoes in G1, cucumbers in G2, and watermelons in G3. There are five types of sensor modules; GPS, beam's signal distance indicator, compass, black&white camera, and color&weight sensor. The robots are using different sensors to estimate their states as shown in Figure 1. The output of the GPS is a pair of numbers (*x*, *y*), 1 ≤ *x*, *y* ≤ 10, indicating the vertical and horizontal positions, and the output of the beams' distance indicator is a pair of numbers (*b*_1_, *b*_2_), 2 ≤ *b*_1_, *b*_2_ ≤ 20, where *b*_1_ and *b*_2_ are the 1-norm distance to the beams. The compass sensor gives the direction of the robot and the other sensor modules are used to distinguish the kind of crops as in Table 1. Robots 1 and 3 use the color&weight sensor and robot 2 uses black&white camera to distinguish the kind of crops as explained in Figure 1.

Consider an abstract robot whose state is a pair (*n*, *k*) ∈ *S*′ where 1 ≤ *n* ≤ 100 is the number of grid when numbering the grids from left to right and bottom to top, and *k* ∈ {to, cu, wa, 0}. The terms to, cu, wa, and 0 represent tomato, cucumber, watermelon, and nothing, respectively. The set of action is the same as the set of actions of robot 1; that is, *A*′ = *A*_1_. One can easily check that there are three homomorphisms *h*_1_, *h*_2_, and *h*_3_ from the MDPs of robots 1, 2, and 3 to the MDP of the abstract robot, relating equivalent pairs of state-action in the MDPs. For example, we have

(7) h13,4,RL,N=33,to,N,h12,1,YH,W=2,wa,W,h25,1,E,T,L=5,cu,0,h210,2,N,0,P=20,0,P,h36,7,S,GL,LF=85,cu,E,h318,8,W,0,F=93,0,W.

Therefore, robots 1, 2, and 3 have the same environment's dynamics and reward and are context transferable, although they do not have the same MDP and there are no one-to-one mappings between their sets of states and actions. This is because of the existence of redundancy in their representations of the environments, which is the case in most practical applications.

## 3. Why Context Transfer Is Important

Most of the current transfer learning approaches in RL are typically framed as leveraging knowledge learned on a source task to improve learning on a related, but different, target task. These approaches are able to successfully transfer knowledge between agents in different tasks.

This paper discusses context transfer in RL, that is, transferring knowledge between agents with different states and action spaces. The goal in this type of transfer problem is the same: reduce the time needed to learn the target with transfer, relative to learning without transfer. We think that this is an important problem for the following reasons.

Firstly, there may be different agents with different sensors or actuators in an environment and cooperation between them may improve the learning process. These agents can be similar to the robots of Example 11. Solving the problem of context transfer can facilitate the cooperation between such agents.

Secondly, in many real-world scenarios, one actually does not have access to a minimized MDP model of the environment, and usually there is a lot of redundancy in the MDP model. In this case, there may be an agent that has already been training on a task with a certain internal representation of the states and actions but the performance is poor. A different internal representation could allow the agent to achieve higher performance. Context transfer enables the agent to use the previous knowledge to accelerate the learning with new state and action spaces.

Thirdly, consider a real-world working learning system. At some point, we decide to upgrade its sensor and/or actuator modules. Any change in these modules will result in a different description of the environment's dynamics and the reward function. Therefore, the learning algorithm and the trained knowledge are no longer applicable. If experience is expensive in the environment, it is preferable to leverage the existing knowledge of the agents to improve the learning with new sensors or actuators. Context transfer can resolve the problem.

To solve the problem, one needs a mapping between state-action spaces of the agents. Taylor et al. [8] use a hand-coded mapping between the states and actions of the source and target tasks, namely *χ*_*S*_ and *χ*_*A*_. The mapping *χ*_*S*_ maps each state variable of the target task to the most similar state of the source task. Similarly, the mapping *χ*_*A*_ maps each action of the target task to the most similar action of the source task. This pair of mappings is called intertask mapping. They use the intertask mapping to transfer the action-value functions from the source to the target task, thus improving the learning of the target task. In [9], the intertask mapping is used to transfer the samples from the source to the target task. In [10], Taylor and Stone use the intertask mapping to transfer the source task policy to the target task as some rules. The transferred rules summarize the source task policy. The intertask mapping acts as a translator for the rules to be used in the target task. In some cases, it is not possible to define the relation of the state-action spaces of the agents by the intertask mapping (a pair of mappings). For instance, in Example 11, one cannot define a direct mapping as a relation between the actions of robots 1 and 2; for example, there is no equivalent action of robot 1 (up, down, left, and right) to the action of “move forward” of robot 2. Although it is possible to define a mapping between the state-action pairs of the robots, when the state of robot 2 is “up” and moves forward, it equals to move “up” of robot 1. Therefore, we use a mapping between the state-action pairs instead of intertask mapping.

Blockeel et al. [11] transfer relational macros among tasks with different state features and actions. In this approach, relational macros are defined as finite-state machines in which the transition conditions and the node actions are represented by first-order logical clauses. The macros characterize successful behavior in the source task. Inductive logic programming is used to learn a macro and then use it in the early learning stages of the target task.

Ravindran and Barto [12], Soni and Singh [13] use the homomorphism framework to map tasks to a common abstract level. The options are defined on an abstract MDP, called relativized options, and their policies are then transformed according to the specific target task. More specifically, a set of possible transformations is provided and the goal of transfer is to identify the most suitable transformation of the relativized options depending on the current target task.

Konidaris and Barto [14, 15] define options at a higher level of abstraction that can be used by the target task without any explicit mapping between the states and actions of the tasks. In this approach, the tasks' similarities are modeled as agent-space and the tasks' differences are modeled as problem-space. The tasks are assumed to share common features and to be reward-linked; rewards are allocated similar to tasks. An agent learns a portable shaping function from experience in the source tasks in the agent-space to improve the performance in the target task. The presented definition of the notion of reward-linked is mostly qualitative rather than a precise mathematical definition.

This paper tries to present a formal definition of the context transfer problem. This definition has some overlap with the mentioned approaches, but its framework and mathematical formulation is given for the first time. We use the notion of MDP homomorphism to exactly formulate context transferable tasks. The presented algorithm to solve the problem does not require an exact intertask mapping or the existence of some shared features between tasks as mentioned in the previous approaches; it only requires a partial mapping between some features of the source and target tasks. It also has the capability of combining the knowledge of several different source tasks to be used by the target task.

## 4. Feature Space as a Translator between Tasks

In [14], the notion of shared features is used for knowledge transfer among tasks. The shared features are used by an agent to learn a portable shaping function in a sequence of tasks to significantly improve performance in a later related task. In this paper, we follow the same idea of using the feature space as a tool of knowledge transfer. However, our problem, its formulation, and the proposed solution are different. Generally, an agent is equipped with a suite of sensors and actuators. The agent senses the state of the environment using the output of the sensors and performs an action using its actuators. The tuple of the outputs of the sensors and actuators is considered as a feature vector. Let the number of the sensors and actuators of task *T*_*i*_ be *n*, and *f*_*j*_ denote the *j*th element of the feature vector. The feature vector is represented by (*f*_1_^*i*^, *f*_2_^*i*^,…, *f*_*n*_^*i*^) ∈ *F* ^*i*^, where *F* ^*i*^ = *F*_1_^*i*^ × *F*_2_^*i*^ ⋯ ×*F*_*n*_^*i*^ is the space of the feature vectors. *F*_*j*_^*i*^ is the set of all feature values of the *j*th feature. This mapping assigns a pair of state-action to every feature vector:

(8) $$\begin{array}{c} L_{i}:F^{i}⟶S_{i}\times A_{i}, \end{array}$$

where *S*_*i*_ and *A*_*i*_ are the set of states and actions of task *T*_*i*_, respectively.

To transfer the knowledge of the source to the target task (which has a different context), one needs some information to relate the *Q*-values of the state-action pairs of the source to the target task. Such information may be uncertain and ambiguous or even not available in some cases. To solve the problem in such situations, we use the domain knowledge of the feature space as some relations between the feature vectors of the source and target tasks. This information can be expressed by a mapping as

(9) $$\begin{array}{c} K_{i}:F^{l}⟶F^{i}. \end{array}$$

**Figure 2:**
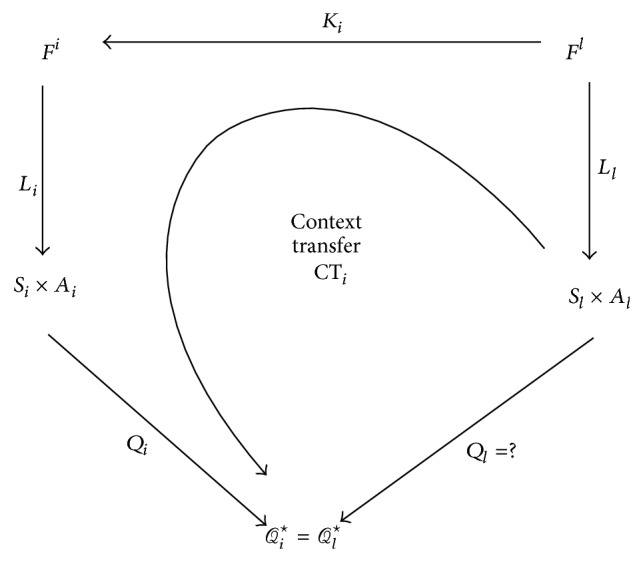
The process of context transfer between source task *T*_*i*_ and target task *T*_*l*_ in which all mappings are known except *Q*_*l*_.

This mapping relates a feature vector of the source task *i* to a feature vector of the target task. Generally, this mapping can be a one-to-many mapping. If this is a one-to-one mapping, then there is an exact correspondence between the feature vectors of the source and target tasks and the knowledge can be transferred between tasks without any ambiguity. The process of context transfer between source task *T*_*i*_ and target task *T*_*l*_ is shown in Figure 2.

In this diagram, the *Q*_*i*_ mapping is the result of the learning process of the source task *T*_*i*_ and assigns an optimal *Q*-value from the set of optimal *Q*-values, *Q*_*i*_^⋆^, as defined in Section 2, to every state-action pair. As the source and target tasks are context transferable, therefore, *Q*_*i*_^⋆^ = *Q*_*l*_^⋆^. The learning process of the target agent estimates the mapping *Q*_*l*_. We use the other mappings to estimate an approximate mapping as CT as an initial estimation of *Q*_*l*_ and thus accelerating the learning process of the target task. This is shown in the diagram of Figure 2. For every (*s* ^*l*^, *a* ^*l*^)∈(*S*_*l*_, *A*_*l*_):

(10) $$\begin{array}{c} {\text{CT}}_{i}\left(s^{l},a^{l}\right)=Q_{i}\circ L_{i}\circ K_{i}\circ L_{l}^{-1}\left(s^{l},a^{l}\right), \end{array}$$

where *G*∘*H*(·) denotes the mapping composition of *G* and *H*, namely *G*∘*H*(·) = *G*(*H*(·)). The mapping *L*_*i*_^−1^ denotes the inverse mapping of *L*_*i*_, and CT_*i*_(*s* ^*l*^, *a* ^*l*^) is an estimation of *Q*_*l*_(*s* ^*l*^, *a* ^*l*^) using context transfer.

**Example 12 .**

Referring to Example 11, suppose that the tasks of robots 2 and 3 are the source and target tasks, respectively. The shared features among tasks are the values of the compass sensor and actuators. We also know that both the values of *c* = 0 for robot 2, and *k* = 0 for robot 3 refers to the value of “Nothing.” We use this information to relate the state-action pairs of the source and target tasks. For instance, we have

(11) K2(3,12,N,RL,F)  =x,y,N,c,F ∣ 1≤x, y≤10, c∈SG,T,BG.

## 5. Knowledge Fusion and Transfer

In [3], the solution methods of RL transfer problems are grouped into five categories; starting-point methods, imitation methods, hierarchical methods, alteration methods, and new RL algorithm methods. In starting-point methods, instead of zero or random initialization in the target task, the target task is initialized based on the knowledge from the source task. Imitation methods involves the transfer methods in which the source-task policy is applied to choose some actions when learning the target task. The third class of RL transfer includes hierarchical methods. These methods view the source as a subtask of the target. The next class of RL transfer methods involves altering the state space, action space, or reward function of the target task based on source-task knowledge. It involves simplifying the state space by state abstraction, decreasing the action space and reward shaping. The new RL algorithm methods consist of entirely new RL algorithms. These approaches address transfer as an inherent part of RL.

In this paper, we adopt a starting-point method to transfer the knowledge; we use the knowledge of the source tasks to initialize the learning of the target task, instead of zero or random initialization. Suppose that the learning of the source tasks are stopped at a certain time because of a learning criteria and the *Q*-values of the source tasks are used by the target task, which is at the initial steps of the learning.

Now, consider the set of *T* = {*T*_1_, *T*_2_,…, *T*_*l*_} where *T*_1_, *T*_2_,…, *T*_*l*−1_ are the source and *T*_*l*_ is the target task as explained in Section 2. The mapping CT_*i*_(*s* ^*l*^, *a* ^*l*^) relates an optimal *Q*-value to state-action pair (*s* ^*l*^, *a* ^*l*^) ∈ *S*_*l*_ × *A*_*l*_. As the mapping *K*_*i*_ may be a one-to-many mapping, therefore, CT_*i*_ is a multivalued function, and CT_*i*_(*s* ^*l*^, *a* ^*l*^) is a set-value instead of being a single value, that is, CT_*i*_(*s* ^*l*^, *a* ^*l*^)⊆*Q*_*l*_^⋆^. For *i* ∈ {1,2,…, *l* − 1}, we will have *l* − 1 different set-values for a state-action pair of the target task. One can easily combine the knowledge of different source tasks using the intersection operator on the *l* − 1 set-values, as

(12) $$\begin{array}{c} \text{CT}\left(s^{l},a^{l}\right)=\underset{i\in \{1,\ldots ,l-1\}}{⋂}{\text{CT}}_{i}\left(s^{l},a^{l}\right). \end{array}$$

This is the set of possible *Q*-values for the pair of (*s* ^*l*^, *a* ^*l*^) using the knowledge of the source tasks. These definitions are used to initialize the *Q*-values of the target task. We can use a statistical average operator to estimate a single value from the set-value CT(*s* ^*l*^, *a* ^*l*^) as an initial value of *Q*_*l*_(*s* ^*l*^, *a* ^*l*^). For example, we can use* mean*,* median*, or* midrange* operators. In this paper, we use the* midrange* operator, defined as follows:

(13) $$\begin{array}{c} \tilde{Q_{l}}\left(s^{l},a^{l}\right)=\text{midrange}\left(\text{CT}\left(s^{l},a^{l}\right)\right), \end{array}$$

where $\tilde{Q_{l}}(s^{l},a^{l})$ is an initial estimation of *Q*_*l*_(*s* ^*l*^, *a* ^*l*^) and

(14) midrangex1,x2,…,xk  =max⁡(x1,x2,…,xk)+min⁡(x1,x2,…,xk)2.

This operator has some advantages over the* mean* or* median* operators because of some implementation issues. There are also some intuitive explanations for using this operator as discussed in the next section.

## 6. *Q*-Intervals for Knowledge Fusion

As explained in the previous section, the context transfer mapping, CT_*i*_, usually is a one-to-many mapping. Therefore, the value of CT_*i*_(*s* ^*l*^, *a* ^*l*^)⊆*Q*_*l*_^⋆^ and (*s* ^*l*^, *a* ^*l*^)∈(*S*_*l*_, *A*_*l*_) is a set-value rather than a single-value. We use an interval-based approach to represent the uncertainty of this set-value. Consider the following definition:

(15) $$\begin{array}{c} IQ_{i}\left(s^{l},a^{l}\right)=\left[Q_{i}^{-}\left(s^{l},a^{l}\right),Q_{i}^{+}\left(s^{l},a^{l}\right)\right], \end{array}$$

where

(16) $$\begin{array}{c} Q_{i}^{-}\left(s^{l},a^{l}\right)=\underset{Q^{⋆}\in {\text{CT}}_{i}\left(s^{l},a^{l}\right)}{\mathrm{Min}}Q^{⋆},\\ Q_{i}^{+}\left(s^{l},a^{l}\right)=\underset{Q^{⋆}\in {\text{CT}}_{i}(s^{l},a^{l})}{\mathrm{Max}}Q^{⋆}. \end{array}$$

We call it a *Q*-interval. To every *Q*-interval, two measures are related as

(17) CntrQ−,Q+=Q−+Q+2,UncrQ−,Q+=Q−−Q+.

These measures are called* center* and* uncertainty* measures of the *Q*-interval, respectively. Let [*Q*_1_^−^, *Q*_1_^+^] and [*Q*_2_^−^, *Q*_2_^+^] be two *Q*-intervals. These definitions induce two orderings on the *Q*-intervals, as follows:

(18) Q1−,Q1+≤RQ2−,Q2+⟺Q1−≤Q2−,Q1+≤Q2+,Q1−,Q1+≤KQ2−,Q2+⟺Q1−≤Q2−,Q2+≤Q1+.

The first ordering can be understood as more rewarding, and the second one can be considered as more accurate ordering. These orderings can induce a mathematical bilattice structure [16–18] on the set of *Q*-intervals. In [19, 20], bilattices are discussed as a tool of reasoning about knowledge when multiple agents are present. Consider the following definitions.

We use the following operators to combine the knowledge of different agents:

(19) $$\begin{array}{c} \left[Q_{1}^{-},Q_{1}^{+}\right]⊕\left[Q_{2}^{-},Q_{2}^{+}\right]=\left[\min\left(Q_{1}^{-},Q_{2}^{-}\right),\max\left(Q_{1}^{+},Q_{2}^{+}\right)\right]. \end{array}$$

This operator is called gullibility in bilattices.

One can easily show that the set-values of context transfer mapping and their corresponding *Q*-intervals have the following properties:

(20) midrangeCTisl,al=CntrIQisl,al,CTisl,al⊆CTjsl,al⟹IQisl,al≤KIQjsl,al,CTksl,al=CTisl,al∩CTjsl,al⟹IQksl,al=IQisl,al⊕IQisl,al.

There are some intuitive explanations for these relations, as well. For example, the second relation says that as the set-value goes to a single-value, the corresponding *Q*-interval will go to an exact value and represent higher-ordered knowledge. The third relation has the same intuition as the gullibility operator of the bilattices. Therefore, we just need to record the minimum and maximum values of the set-values (or the corresponding *Q*-interval) and use the following relation to combine the knowledge of different source tasks:

(21) $$\begin{array}{c} \tilde{Q_{l}}\left(s^{l},a^{l}\right)=\text{Cntr}\left(\underset{i\in \{1,2,\ldots ,l-1\}}{⨁}IQ_{i}\left(s^{l},a^{l}\right)\right). \end{array}$$

## 7. Case Studies and Results

To verify the validity of the proposed algorithms, two case studies are considered. We discuss these case studies in the next sections.

### 7.1. Agents with Different Sensors or Actuators

There are some agents in an environment doing the same tasks even as using different sensors or actuators. Cooperation or knowledge transfer between these agents can improve the learning. These agents can share their knowledge using context transfer. Consider the robots of Example 11; let the robots 1 and 2 be the source and robot 3 be the target agent. Suppose that the farm is a 50 × 50 grid with some randomly located puddles. The reward function is as follows:

(22) Reward=−1Taking  an  action  except  the  following−10Entering  a  puddle,wrong  pickup  or  dropoff100Reaching the goal.

The source agents (robots 1 and 2) have learned their task for 1000 episodes. To examine the algorithm, the learning process of the target task is run four times; first without any transfer, second with transfer from robot 1, third with transfer from robot 2, and forth with transfer from both robots 1 and 2 after knowledge fusion.

The target agent learns for 500 episodes, and the whole learning is repeated for 50 times. The action selection policy is softmax, and the learning parameters are as follows; the learning rate (*α*) is 0.1, the discount factor (*γ*) is set to 0.9 and the temperature (*τ*) decreases by the exponential function (*τ* = *e* ^−0.1*n*^ + 0.5) where *n* is the number of episodes.

**Figure 3:**
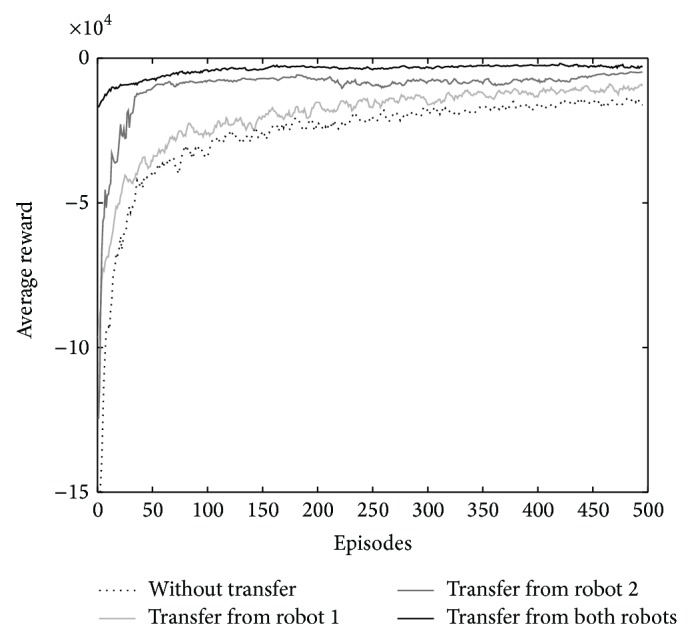
The comparison of average reward of learning for the four cases of transfer: without transfer, with transfer from robot 1, with transfer from robot 2, and with transfer from both robots.

**Figure 4:**
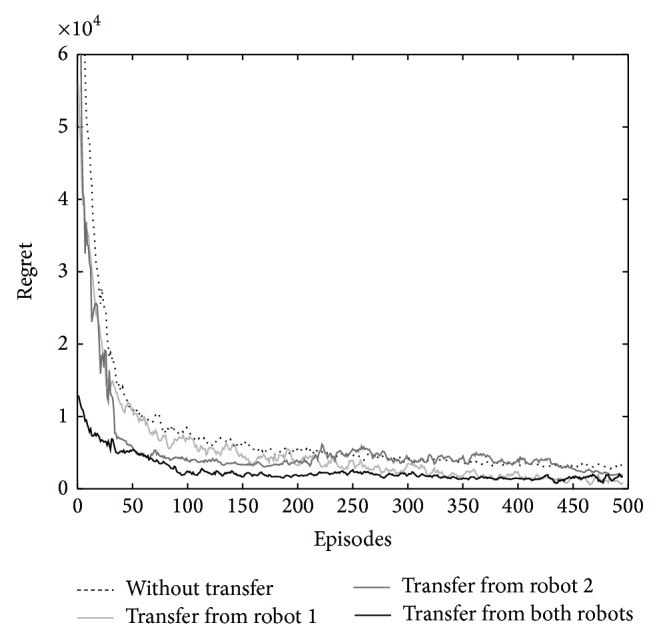
The comparison of regret of learning for the four cases of transfer: without transfer, with transfer from robot 1, with transfer from robot 2, and with transfer from both robots.

The final learning curves are averages of 50 independent learning curves. The average reward and regret function of the learning are shown in Figures 3 and 4, respectively. The regret is the expected decrease in reward because of executing the algorithm instead of acting optimally from the beginning [21]. The results show the increase of average reward and decrease of regret at the beginning episodes of the learning. The knowledge fusion of robots 1 and 2 significantly improves the learning.

### 7.2. Changes in the Sensory-Motor System of a Learning Agent

Representation is one of the key components of any reinforcement learning algorithm. Any change in the representation will result in a different description of the environment's dynamics and the reward function, and the learning algorithm is no longer applicable. The reinitialization of the whole learning process is undesirable, especially, when the experience is expensive. For example, upgrading the sensory-motor system of an agent even as saving the previous knowledge. Context transfer from the task with old sensory-motor system to the task with new sensory-motor system can solve this problem.

**Figure 5:**
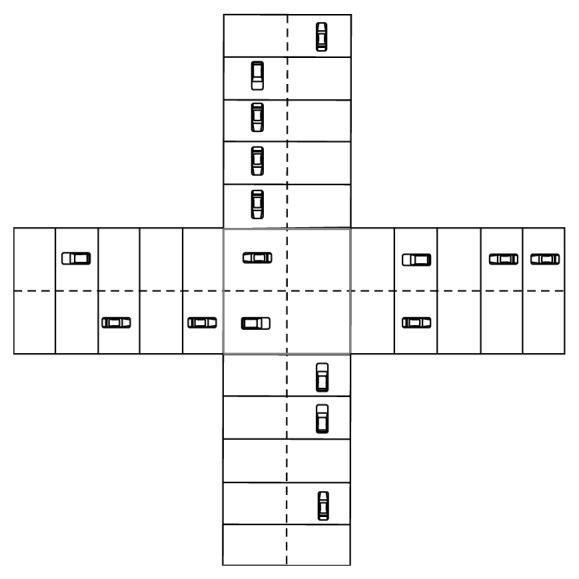
Crossroad Traffic Controller. Old system: Sensors: distance sensor, *S*_old_ = {(*x*, *y*, *d*) | 1 ≤ *x*,  *y* ≤ 10,  *d* ∈ {*V*, *H*}}, *x*: distance to first car in vertical lane, *y*: distance to first car in horizontal lane, *V*: vertical lane is green, *H*: horizontal lane is green, *A*_old_ = {*GV*, *GH*, *N*}, *GV*: change the vertical lane to green, *GH*: change the horizontal lane to green, *N*: no action. New system: Sensors: camera, *S*_new_ = {(*x*, *y*, *d*) | 0 ≤ *x*,  *y* ≤ 1023,  *d* ∈ {*V*, *H*}}, *x*: cars' existence coding in the first ten squares of the vertical lane, *y*: cars' existence coding in the first ten squares of the horizontal lane, *V*: vertical lane is green, *H*: horizontal lane is green, *A*_new_ = {*C*, *N*}, *C*: change the light, *N*: no action.

We use the “Crossroad Traffic Controller” task as an example. This problem is a modified version of the scenario discussed in [22], which is a stochastic task. There is a crossroad with two-way road resulting in a four-square grid at the center, a horizontal and a vertical one. The task is to control the traffic light by switching the green light between the vertical and horizontal lanes to keep the queues in front of the traffic light as small as possible (Figure 5). In front of the light of each lane, only five squares are considered. The reward is the total amount of cars in front of the traffic light times −1. Switching the light causes a transition period of 4 time steps in which one traffic light is orange and the other one is red. During this transition period no cars can pass the crossroad and actions taken in this period have no effect. The speed of the cars is one square per time step.

The system is working with an old sensor that gives the distance to the first car approaching the crossroad in each lane. Therefore, the old system has 5 × 5 × 5 × 5 × 2 states as described in Figure 5. The aim is to upgrade the sensor of the system to a sensor that gives the existence of cars in each square. The actions of the system are also changed as shown in Figure 5. We use the relation between features of the old and new sensors to transfer the knowledge.

Passing of 100 cars is considered as an episode of learning. The learning is repeated for 40 000 episodes. The action selection policy is softmax and the learning parameters are as follows; the learning rate (*α*) is 0.1, the discount factor (*γ*) is set to 0.9 and the temperature (*τ*) decreases by the exponential function (*τ* = 5*e* ^−0.1*n*^ + 0.5) where *n* is the number of episodes.

**Figure 6:**
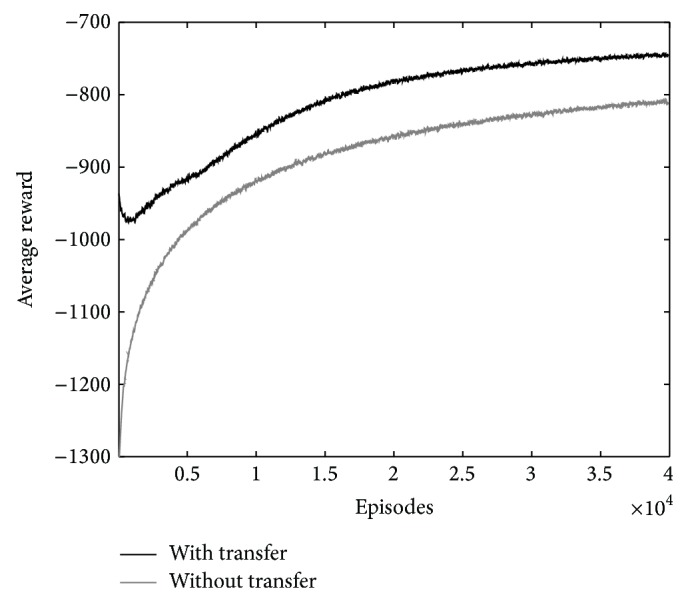
The comparison of average reward of learning with and without transfer for crossroad traffic controller.

**Figure 7:**
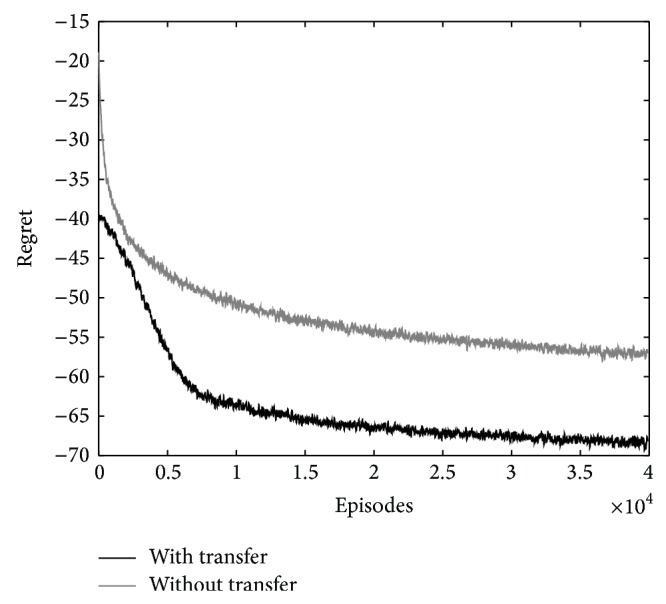
The comparison of regret of learning with and without transfer for crossroad traffic controller.

The final learning curves are averages of 50 independent learning curves and are shown in Figures 6 and 7. Smoothing is performed on the curves with a moving window average for better representation. The length of the window is 50 episodes. The results show the increase of average reward and decrease of regret of the learning when using knowledge transfer.

## 8. Conclusion

Transfer learning in heterogeneous RL tasks is a challenging area. The heterogeneity between tasks may be because of the difference between state-action spaces and transition models of the environment or reward functions. Context transfer, as defined in this paper, discusses knowledge transfer between tasks with different state-action spaces. The tasks with the same environment's dynamics and reward function but with different state-action spaces were called context transferable tasks. The problem was formulated in terms of MDP homomorphism. It was shown that the context transferable tasks have the same set of optimal action values. The feature space was used as a translator between different tasks to transfer the knowledge from the source to the target tasks. An interval-based approach was used to represent and combine the knowledge of the source tasks. The proposed knowledge transfer approach was tested in two different case studies. The results show the effectiveness of the proposed approach.

## Figures and Tables

**Table 1:** The output of the sensor module for different kinds of crops.

Crops	B&W camera	Color	Weight
Tomato	Small globe	Red	Light
Cucumber	Rod	Green	Light
Watermelon	Big globe	Green or yellow	Heavy
